# A Rare Surgical Case of Gallbladder Volvulus Masquerading as Acute Cholecystitis

**DOI:** 10.7759/cureus.92223

**Published:** 2025-09-13

**Authors:** Brandon Velazquez, Konstantina Kostara, Jacob Seltzer, George Tsioulias, Zbigniew Moszczynski

**Affiliations:** 1 Surgery, CarePoint Health, Bayonne, USA

**Keywords:** abdominal pain, acute cholecystitis, biliary surgery, case report, diagnostic challenge, elderly patient, gallbladder ischemia, gallbladder volvulus, laparoscopic cholecystectomy, percutaneous cholecystostomy

## Abstract

Gallbladder volvulus (GBV) is a rare and life-threatening condition characterized by the twisting of the gallbladder on its pedicle, resulting in obstruction of the cystic duct, artery, and vein. This leads to compromised blood flow, ischemia, and potentially severe complications, including necrosis or perforation. Although GBV shares clinical symptoms with acute cholecystitis, it requires urgent surgical intervention due to its rapid progression and severe consequences if left untreated. GBV predominantly affects elderly females and is often associated with anatomical variations that allow for increased mobility of the gallbladder, contributing to its torsion. An 84-year-old female presented with 24 hours of severe, diffuse abdominal pain, nausea, and vomiting. Initial imaging suggested cholecystitis, prompting the placement of a percutaneous cholecystostomy tube. However, laparoscopic cholecystectomy revealed a torsed, hyperemic gallbladder with signs of ischemia, though no perforation was noted. The gallbladder was detorsed, and the cystic duct and artery were divided. The patient recovered uneventfully and was discharged on postoperative day one.

GBV often presents with clinical features similar to acute cholecystitis, complicating diagnosis. Imaging, including ultrasonography and CT scans, may reveal thickened gallbladder walls and pericholecystic fluid, but the absence of gallstones and identification of atypical anatomical positioning can raise suspicion for GBV. Surgical treatment, particularly laparoscopic cholecystectomy, remains the gold standard for management. Early recognition and intervention are crucial to prevent gallbladder necrosis, perforation, and biliary peritonitis. This case underscores the importance of heightened clinical suspicion and timely intervention to reduce morbidity and mortality associated with GBV. GBV is a rare but serious cause of acute abdominal pain, particularly in elderly patients. This case highlights the diagnostic challenges and the critical role of early intervention in preventing severe complications. Accurate imaging and surgical treatment are essential for successful outcomes.

## Introduction

Gallbladder volvulus (GBV) is a rare and life-threatening surgical emergency characterized by the twisting of the gallbladder around its pedicle, leading to obstruction of the cystic duct, artery, and vein [1]. This torsion results in compromised blood flow, ischemia, and, if untreated, severe complications such as necrosis, gangrene, or perforation [2]. GBV is an exceptionally uncommon condition, with an estimated incidence of one in 365,000 cases of gallbladder disease [3]. First described by Wendel in 1898, GBV remains a diagnostic challenge due to its non-specific clinical presentation, which often mimics acute cholecystitis [4].

The typical clinical manifestations of GBV include sudden onset of severe right upper quadrant (RUQ) pain, fever, nausea, and vomiting, often accompanied by leukocytosis [1]. However, unlike acute cholecystitis, the pain associated with GBV is more intense, has a sharper onset, and may be accompanied by signs of peritoneal irritation [5]. The rapid progression of GBV makes early recognition and prompt surgical intervention critical to prevent complications such as sepsis, bile peritonitis, and multiorgan failure [2].

GBV predominantly affects elderly females, with a female-to-male ratio of approximately 3:1, possibly due to increased gallbladder mobility resulting from age-related loss of visceral fat and liver atrophy [6]. Interestingly, in pediatric cases, GBV appears to be more common in boys than girls [7]. Several anatomical factors predispose individuals to GBV, including a long mesentery, an atrophic liver, and the absence of peritoneal or connective tissue attachments to the gallbladder [6]. These anatomical variations create a “floating gallbladder,” allowing it to twist freely around the cystic duct and artery [1].

Due to its rarity and the overlap of symptoms with more common biliary diseases, GBV is often misdiagnosed preoperatively, with accurate diagnosis occurring in fewer than 10% of cases [5]. Imaging studies, such as ultrasound and computed tomography (CT), may reveal an abnormally positioned gallbladder with signs of ischemia, but a definitive diagnosis is frequently made intraoperatively [3]. The presence of the “whirl sign” on CT, indicating twisted cystic structures, is a key radiologic feature that may aid in preoperative diagnosis [4]. Early surgical intervention, typically via laparoscopic cholecystectomy, is essential to prevent catastrophic outcomes [5].

Given the low preoperative diagnostic rate and the potential for rapid clinical deterioration, clinicians must maintain a high index of suspicion for GBV in patients presenting with atypical acute cholecystitis, especially in elderly females or those with predisposing anatomical factors [6]. Raising awareness of this rare condition and its distinguishing clinical and radiological features is crucial for improving early recognition and patient outcomes [1].

Given these diagnostic challenges, this study aimed to describe the presentation, imaging findings, and surgical management of gallbladder volvulus in an elderly female patient, highlighting key clinical features that may aid in earlier recognition and differentiation from acute cholecystitis.

## Case presentation

**Figure 1:**
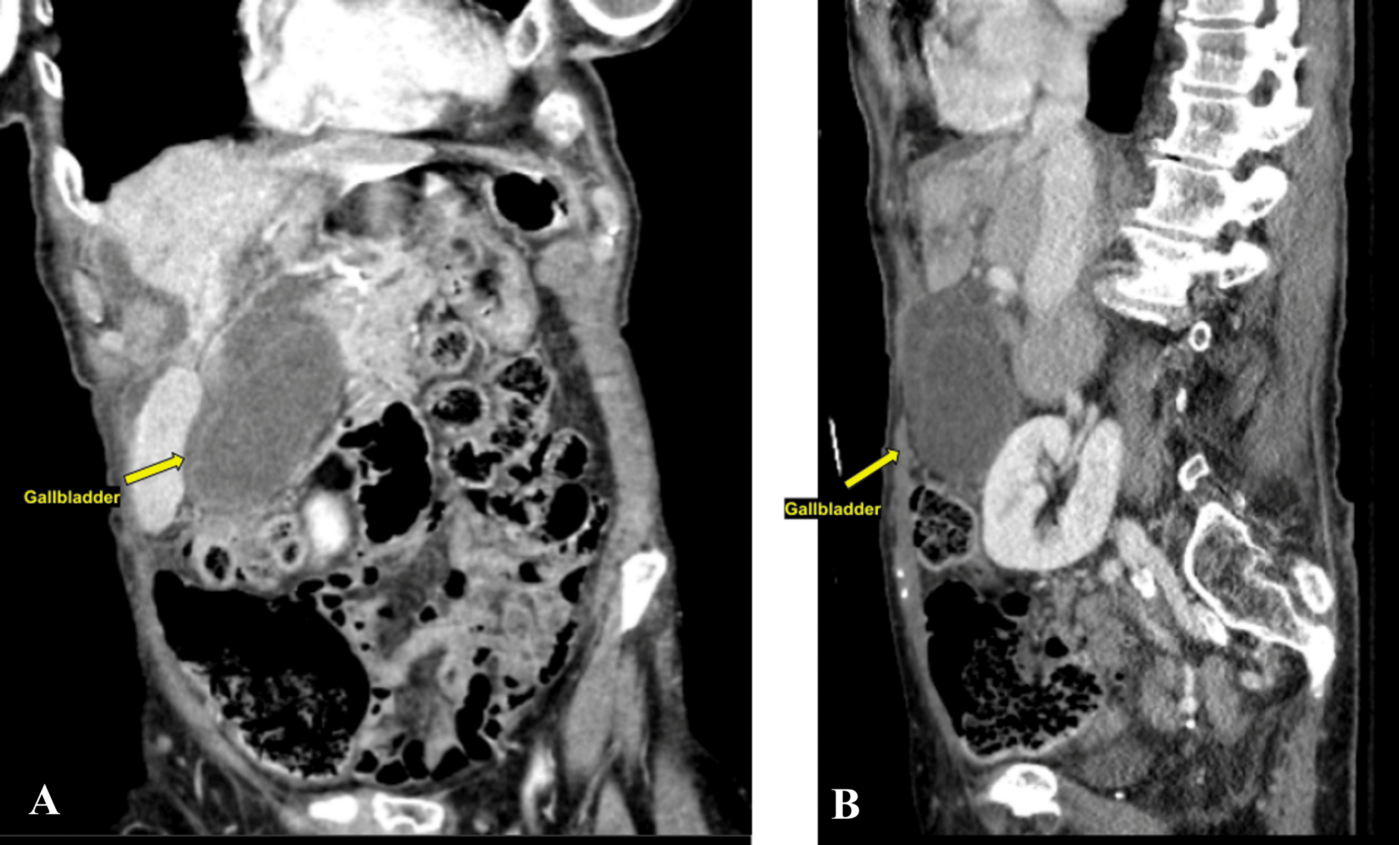
CT scan with IV contrast demonstrating a markedly distended gallbladder with wall thickening, pericholecystic fluid and fat stranding (left: coronal view, right: sagittal view). (A) axial view of computed tomography, arrow indicates the distended gallbladder. (B) sagittal view of computed tomography, arrow indicates the distended gallbladder. The classic findings (“whirl sign,” “floating gallbladder,” etc.) were not seen.

**Figure 2:**
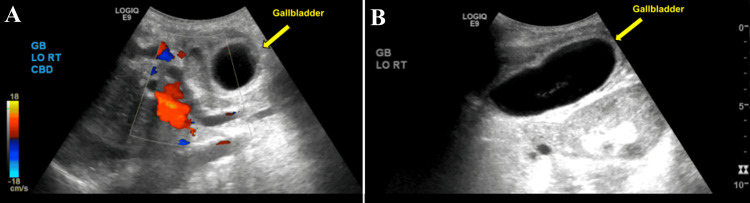
Right upper quadrant ultrasound demonstrating a distended gallbladder with a thickened wall of 4.2 mm, pericholecystic edema, biliary sludge, and absence of gallstones. (A) Short-axis view of the ultrasound, arrow indicates the distended gallbladder. (B) Long axis view of the ultrasound, arrow indicates the distended gallbladder.

This is a case of an 84-year-old female who presented to the emergency department with 24 hours of sudden-onset severe diffuse abdominal pain, described as sharp, constant, non-migrating, and non-radiating. The pain was associated with intractable nausea and vomiting; however, the patient continued to have bowel function with formed stool and flatus. The patient was unable to describe any inciting events and could not correlate food intake with the onset of symptoms. She denied prior episodes of similar pain and denied postprandial biliary colic. Upon initial presentation, the patient was found to be afebrile, tachycardic, and hypertensive with systolic blood pressure >200 mmHg. Routine blood tests were normal with the exception of mild leukocytosis, 11.2 × 10⁹/L (normal range 4.5-11 × 10⁹/L), and neutrophilia at 90% (normal range 40-60%). On physical examination, the patient was found to be diffusely tender, with voluntary guarding and rebound tenderness in all four quadrants of the abdomen. A computed tomography (CT) scan of the abdomen and pelvis with intravenous contrast showed a significantly distended gallbladder with a thickened wall as well as surrounding edema and fat stranding (Figures 1A, 1B). A right upper quadrant ultrasound revealed a thickened gallbladder wall of 4.2 mm with wall edema and sludge within a very distended gallbladder, with the common bile duct measuring 8.1 mm, considered normal for her age (Figures 2A, 2B). The findings of the imaging studies confirmed the diagnosis of cholecystitis.

**Figure 3:**
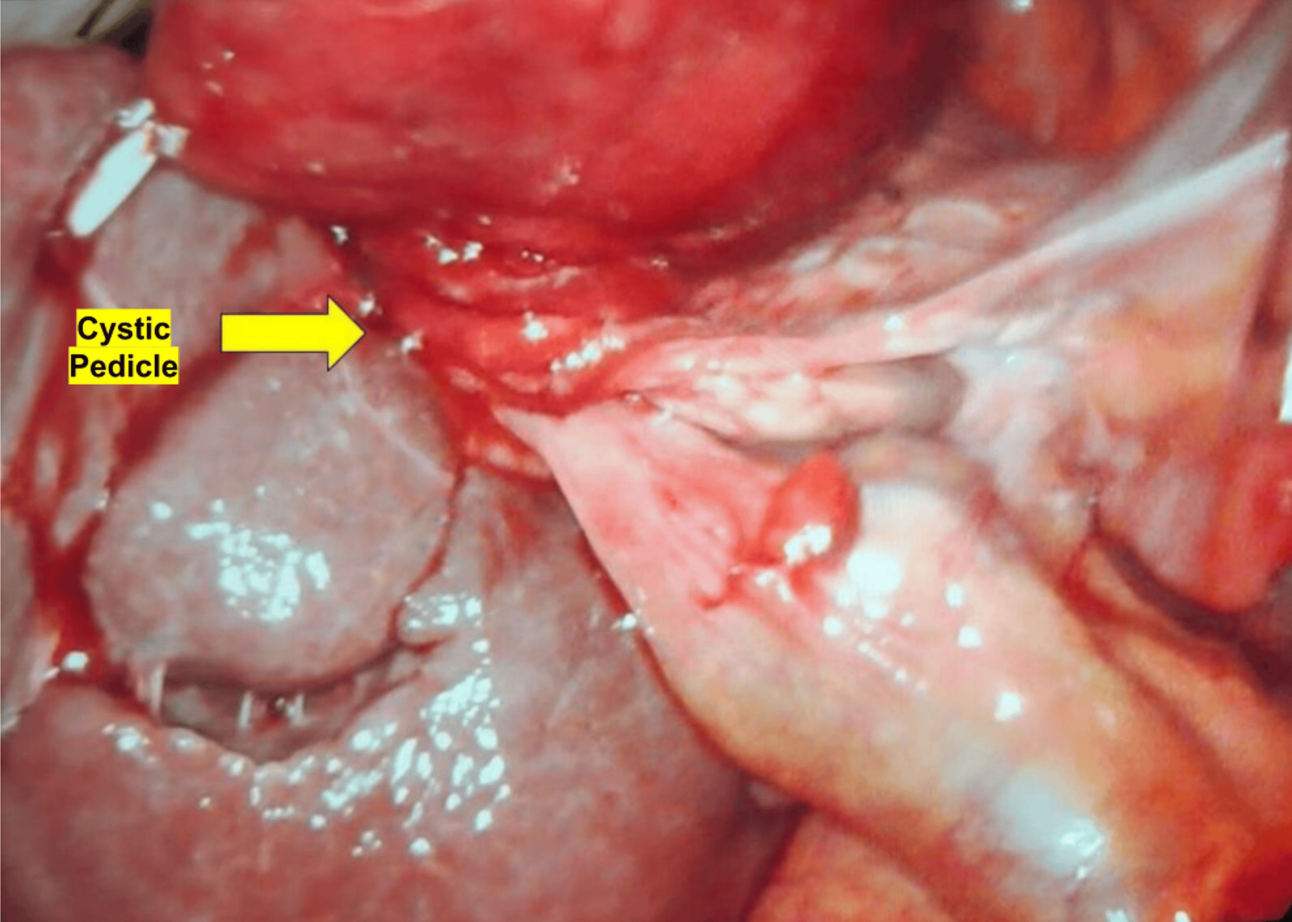
Intraoperative findings shows a grossly distended, hyperemic gallbladder which was torsed along a long pedicle consisting of the cystic duct and cystic artery.

**Figure 4:**
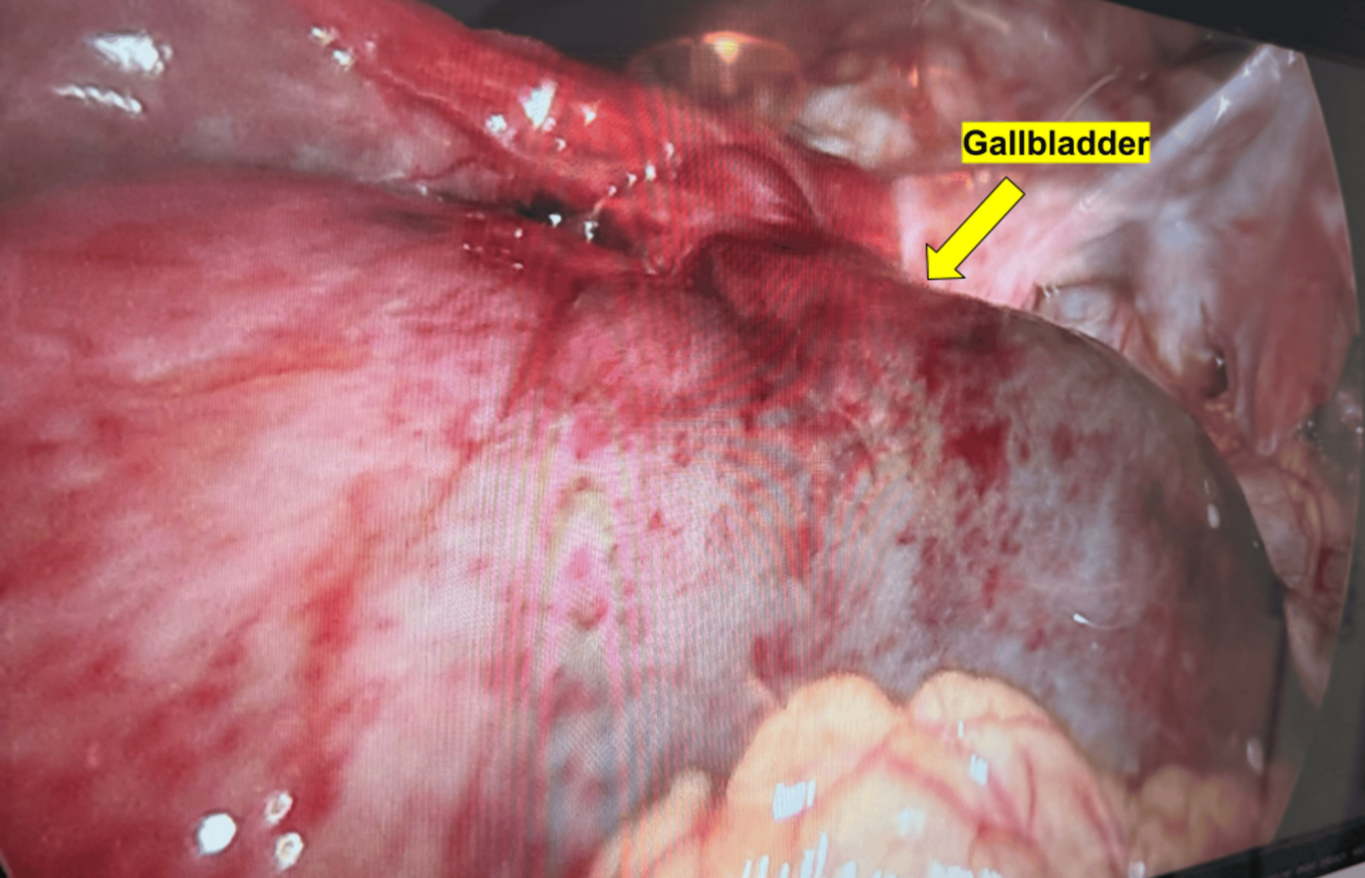
Intraoperative findings shows a significantly distended and hyperemic gallbladder.

A temporizing percutaneous cholecystostomy tube was expeditiously inserted to avert an imminent gallbladder perforation, and shortly thereafter, the patient was taken to the operating room for laparoscopic cholecystectomy. Intraoperative findings were notable for a grossly distended, hyperemic gallbladder, which was torsed along a long pedicle consisting of the cystic duct and cystic artery. The gallbladder was almost completely detached from the liver bed, appearing to be “free floating” (Figures 3, 4). After untwisting the gallbladder counterclockwise, the cystic artery and duct were carefully dissected. The cystic duct was found to be 1.5 cm wide and was divided with a laparoscopic linear cutter stapler. The cystic artery was found to be obliterated and was divided with the laparoscopic bipolar energy device. The surgery was otherwise uneventful, and the patient tolerated it well. Postoperatively, the patient’s pain rapidly subsided, and the vital signs normalized. She was discharged on postoperative day one.

## Discussion

Gallbladder volvulus is a rare condition, and its presentation can be indistinguishable from that of acute cholecystitis. Patients typically present with an acute onset of right upper quadrant pain, nausea, vomiting, and, in some cases, signs of peritonitis. In this case, the unusually severe and diffuse abdominal pain was suggestive of a potentially atypical intra-abdominal process. Fever and leukocytosis are non-specific findings that can further confound the diagnosis. This overlap in clinical presentation with more common biliary diseases underscores the diagnostic dilemma, as initial imaging studies may not clearly reveal the volvulus [1]. Ultrasonography, which is frequently the first-line imaging modality, may only show gallbladder wall thickening or pericholecystic fluid, similar to acute cholecystitis. However, the absence of gallstones in combination with signs of acute inflammation should raise the suspicion of an alternative diagnosis.

Accurate preoperative diagnosis of gallbladder volvulus remains challenging, with computed tomography (CT) and magnetic resonance imaging (MRI) emerging as valuable tools in identifying this condition. Specific imaging findings that suggest gallbladder volvulus include a distended gallbladder, the "whirl sign" representing the twisted cystic duct, and abnormal positioning of the gallbladder outside its usual anatomical location. In the present case, the CT scan did not show a whirl sign, but did show a massively distended gallbladder.

Surgical treatment is the definitive management for gallbladder volvulus, with laparoscopic cholecystectomy being the gold standard. Prompt surgical intervention is critical to avoid gallbladder necrosis, perforation, and subsequent biliary peritonitis [2]. In the present case, the patient underwent an urgent percutaneous cholecystostomy tube placement followed by a laparoscopic cholecystectomy. Intraoperatively, the gallbladder was found to be torsed 360 degrees clockwise around its mesentery, with signs of ischemia and patchy necrosis but no signs of perforation. Her recovery was uneventful, and she was discharged on postoperative day one. This outcome emphasizes the importance of early diagnosis and surgical management in preventing complications associated with delayed treatment.

## Conclusions

Gallbladder volvulus (GBV) is a rare but critical condition that can mimic more common biliary diseases, such as acute cholecystitis, leading to potential misdiagnosis. This case highlights the importance of a high index of suspicion, particularly in elderly patients presenting with atypical abdominal pain and no evidence of gallstones. Prompt recognition, aided by imaging studies such as CT scans and MRIs, is crucial for diagnosing GBV, which may otherwise be mistaken for simpler biliary conditions. Surgical intervention, including percutaneous cholecystostomy and laparoscopic cholecystectomy, remains the definitive treatment, preventing life-threatening complications. In this case, early surgical management resulted in a favorable outcome, underscoring the significance of timely diagnosis and intervention in preventing severe morbidity and mortality associated with this rare condition.
